# Retirement Planning and Financial Anxiety among Nigerian Civil Servants: Insights from Social Comparison Theory

**DOI:** 10.3390/bs13050425

**Published:** 2023-05-17

**Authors:** Lawrence Ejike Ugwu, Erhabor Sunday Idemudia

**Affiliations:** 1 Faculty of Humanities, North-West University, Mafikeng 2790, South Africa; erhabor.idemudia@nwu.ac.za; 2 Psychology Department, Renaissance University, Ugbawka P.O. Box 01193, EN, Nigeria

**Keywords:** retirement anxiety, proactive personality, social comparison order, civil servants in Nigeria

## Abstract

The psychological implication of retirement is underemphasised. This study examined the relationship between proactive personality, social comparison, and retirement anxiety among Nigerian civil servants. The study is a cross-sectional design, using proactive personality, social comparison orientation, and Nigerian pre-retirement anxiety scales. Five hundred and eight staff members in government-owned tertiary institutions with five years or less to go until retirement, and at a mean age of 57.47 (SD = 3.02), were surveyed. The study established that a proactive personality negatively predicted retirement anxiety and that civil servants engage in diverse forms of intrapreneurship/entrepreneurship to augment their savings. The study also revealed that social comparison (opinion) mediated the relationship between proactive personality and retirement anxiety (financial preparedness and social alienation). In addition, the study found that social comparison (opinion and ability) mediated the relationship between proactive personality and retirement anxiety (financial preparedness) in a sequential order. The findings suggest that retirees in Nigeria face complex challenges, including financial unpreparedness, social alienation, and uncertainty. The study highlights the importance of understanding the relationship between personality traits, social comparison, and retirement anxiety in order to develop effective interventions and policies that support retirees in Nigeria.

## 1. Introduction

The apprehension of adjusting to a new lifestyle as one approaches retirement age is considered retirement anxiety [1]. This change can be overwhelming, particularly for those who relied on their career as a primary source of income, identity, and purpose. The fear of running out of money, especially if a person did not save enough for retirement, induces the feeling of worry about how they will manage to pay for healthcare, housing, and other expenses without a steady paycheque. This can cause them to feel overwhelmed, stressed, and uncertain about their future. The fulfilment and meaning people obtain from their careers makes them feel they lost a significant part of themselves. This can lead to feelings of isolation, boredom, and depression [2]. The impact on the relationships built over the years at work makes it difficult to accept that their lifestyle will change when they retire. The everyday relationship with family members might become tense as the shrinking of personal space and curtailed independence become a cause for concern.

Globally, civil servants have specific years of work, or an age at which an employee is expected to retire [3]. Therefore, retirement planning typically involves understanding pension benefits and eligibility criteria and deciding when and how to manage retirement income. However, changes to pension schemes and the uncertain economic climate can make it difficult to plan confidently for retirement. This challenge can be of more concern to employees in developing countries such as Nigeria, which have several uncertainties in their economies. In as much as retirement anxiety is considered a normal and understandable concern for every retiree, the concern for Nigerian civil servants is greater [4]. The need to take more proactive steps to plan and prepare for retirement is necessary for Nigerian civil servants to help alleviate anxiety and feel more confident about their financial future.

Nigeria adopted a contributory pension scheme in 2004 [5] that shifted the risk of retirement savings into the hands of the employee. That is, the plan does not apply any formula for a monthly pension payment, but rather uses the accumulated fund in the employee’s retirement savings account (RSA) at the time of retirement. This RSA is based on the income and percentage of saved income. The employer and the employee contribute a percentage of the employee’s monthly payment to the employee’s retirement benefit fund. This means that a low-income earner could have low retirement savings if they rely on this option alone [6].

The challenges before most people in this category are low savings, volatile financial markets, a rapid population growth, which increases the pressure on pension systems for support, and a lack of political will to implement global agreements entered into by the country to protect the ageing population by providing adequate social security to individuals beyond retirement [7]. In addition, a lack of financial literacy contributes to the struggle faced by an average civil servant. 

Several studies aimed to distinguish the characteristics of individuals who are self-assured in their retirement preparations and believe they are sufficiently equipped, from those who are less confident. While there is a recognised correlation between sociodemographic factors and retirement adequacy, individual psychological traits are also deemed significant.

According to recent studies [8,9,10], individuals with positive financial attitudes and behaviours are more likely to perceive themselves as adequately prepared for retirement (Joo and Pauwels, 2002). Those with longer-term planning perspectives are also more confident about their retirement preparations [10], and those with higher levels of financial risk tolerance are more confident [11]. Furthermore, studies confirmed that individuals who take proactive steps to implement financial knowledge are more likely to be confident in their retirement preparations [12,13], and those who subjectively assess themselves as financially knowledgeable are also more likely to feel adequately prepared for retirement [14,15].

However, a study comparing American and Dutch respondents revealed that a future time perspective was a statistically significant predictor of retirement savings adequacy for Americans but not for the Dutch [15]. Despite these findings, it is still uncertain whether these relationships between behavioural characteristics and retirement confidence exist in developing African countries.

Given the current structure of the Nigerian retirement system and pension scheme, which is limited to the worker’s meagre income, it is crucial to understand how individuals take proactive measures independent of the government for their retirement preparations and factors associated with their perceived retirement adequacy. However, this may differ from countries with reliable public pension plans that provide an additional safety net during retirement [11]. 

There is limited research regarding both financial and psychological retirement adequacy. Many studies investigate the material needs required for effective retirement, forgetting the psychological inadequacies of regrets of not meeting their life expectations, or fear of isolation and loneliness. There is also a paucity of studies on efficient proactive measures adopted to mitigate retirement anxiety among retirees in Nigeria. It is suggested by [16] that individuals experience death anxiety because of their awareness of their mortality and that retirement can exacerbate this anxiety by increasing feelings of isolation and disconnection. This study explores some of these needs and how some psychological investments can mitigate the various dimensions of anxiety that come with retirement.

Studies in Nigeria established that workers engage in intrapreneurship/entrepreneurship within their offices to augment their finances [17,18,19]. Some seek to improve their financial literacy [20], whereas others seek to improve the quality of their health before retirement [21]. Moreover, some are bent on strengthening social networks [22]. Evidently, these are proactive measures workers are involved in to survive the inadequacies in their work life. 

Some people rely on the support they hope to receive from their children when they grow older and begin working [23]. All these are safety measures believed to support retirement plans that are meant to mitigate the fears of retirement.

Studies discovered that proactive behaviour can significantly impact retirement planning [22,24]. Retirement planning involves making decisions about saving, investing, and budgeting to ensure financial security in retirement. By taking a proactive approach, individuals can ensure that they are well-prepared for retirement and have a plan to meet their financial goals.

Greenwood [25] posits that proactive individuals often start planning for retirement early in their careers. This allows them to use the power of compound interest to grow their savings over time. Proactive individuals set clear retirement goals and develop plans to achieve them. This may involve setting a target retirement age, estimating retirement expenses, and determining how much they need to save to achieve their goals [26].

Individuals who review their retirement plans and adjust them based on prevailing circumstances ensure they stay on track to meet their goals despite changing circumstances [16]. They achieve this by seeking the advice of financial planners or other professionals to help them develop a retirement plan and make informed investment decisions [27].

**H1.**  *Proactive behaviour will negatively predict retirement anxiety.*

## 2. Social Comparison and Proactive Behaviour

The process of evaluating oneself and the circumstance in which one finds oneself can be termed self-reflection. This is necessary as one considers retirement. This self-evaluation does not happen in isolation, but in comparison with others. The process of evaluating the future self can be assessed effectively when comparing how those who pursued that route fared. This means that people compare themselves at different levels with others.

Social comparison is the act of utilising other people as sources of information to assess how we are doing compared to them (ability comparison) or to determine how we should behave, think, and feel (opinion comparison) [28]. These comparisons can offer insight into our abilities, social status, and performance, which can help us navigate the social world. Additionally, knowledge about others can satisfy fundamental human needs, such as the need for affiliation and esteem [28]. Social comparison is pervasive across cultures [29] and is present in children [30]. It was also proposed as a critical aspect of human social evolution [31].

The social comparison process centres on selecting the comparison target (upward: someone superior to oneself, versus downward: someone inferior to oneself), and likewise, the consequence of the comparison (assimilation versus contrast). Assimilation happens when the comparer’s self-evaluation moves towards the comparison target, becoming more optimistic after an upward comparison and negative after a downward comparison. Contrast occurs when the comparer’s self-evaluation moves away from the comparison target, becoming more negative after an upward comparison and more positive after a downward comparison. Although people also make non-diagnostic comparisons with irrelevant comparison targets [32], social comparisons are more probable when the comparison dimension is relevant to the self and when the comparison target is similar to the self. For example, in recent meta-analytic research, Ref. [33] investigated studies of over 60 years showed that individuals usually compare themselves to those who outperform them contrastingly, resulting in decreased self-evaluations, envy, and a generally worse mood. 

In a study investigating cultural differences in social comparison, Ref. [34] established that individuals in countries with collectivistic cultures tend to engage in social comparison more frequently than those in individualistic cultures. In their study, participants from Asian countries, which tend to have more collectivistic cultures, reported higher social comparison frequencies than those from Western countries. Nigeria falls under the collectivist culture [35] and usually uses social comparison in making decisions that affect the future. 

According to [36], studies indicated that monetary saving behaviour is determined by long-term income, which is influenced by a person’s life cycle and the ageing process. Financial literacy is another individual-level factor that affects savings, with a positive correlation between understanding financial concepts and the ability to save [37]. Financial education programmes were suggested to make an improvement in saving and financial decision-making, particularly among low-income and poor individuals who may lack sufficient information to make sound financial decisions compared to high-income earners [38]. Goal setting was also perceived to positively impact savings behaviour, as helping people identify a specific goal increases the likelihood of developing a savings habit [39]. Gender was also identified as a primary variable in explaining savings behaviour. Single female households are less likely to save than single male households due to lower levels of income, wealth, and more conservative investment decisions [40,41].

Additionally, access to health insurance was shown to positively affect savings behaviour, with individuals who have health insurance more likely to save compared to similar families without health insurance [39]. Furthermore, the image that financial institutions display also plays a vital role in shaping consumers’ financial behaviour [42,43,44]. Finally, Ref. [36] suggests that most people make decisions based on the shared experiences of those around them. This either encourages or discourages engagement in a particular course of action. It can be argued that this is more effective in a collectivist society such as Nigeria than in an individualistic one.

**H2.**  *Proactive behaviour will positively predict social comparison.*

## 3. Mediating Role Social Comparison

Social comparison plays a significant role in the anxiety experienced by retirees when they compare their achievements, status, and challenges with their peers, colleagues, family members, fictive kins, friends, etc. [45]. The focus on retirement anxiety is pertinent because of the dearth of studies on the intervening role of social comparison in the relationship between proactive behaviour and retirement anxiety. 

Social comparison theory (SCT) proposes that individuals evaluate their abilities and opinions by comparing themselves to others [46]. For example, in retirement, individuals may compare themselves to others who retired or are about to retire to assess how they are doing in comparison. However, this comparison can increase anxiety if the individual perceives himself as not measuring up to his peers.

Thus, a social comparison may mediate the relationship between proactive personality and retirement anxiety. Specifically, individuals with a proactive personality are more likely to engage in upward social comparison (comparing themselves to those who are more successful) and perceive themselves as doing well in comparison. This perception may then lead to decreased retirement anxiety.

Overall, social comparison theory provides a framework to understand how social comparison can influence retirement anxiety and how a proactive personality may shield a person against this anxiety by engaging in upward social comparison.

Social comparison can create unrealistic expectations and standards [47,48], and the same could apply for retirement. Retirement is often portrayed as a time of leisure, relaxation, and freedom from work-related stress [49]. However, these expectations are only sometimes realistic or attainable for everyone. Social comparison can lead individuals to comparing their own retirement plans and lifestyles with others with different financial, social, and health situations. This can result in inadequacy, fear of not meeting the desired standard, and anxiety about the future. Social comparison can also lead to envy or jealousy towards others who may appear to have better retirement plans, thereby increasing anxiety and stress [50].

Additionally, social comparison can impact retirement decisions [51]. Proactive individuals can make better retirement decisions by adjusting their expectations and lifestyle. For example, when individuals perceive that their peers are working for more years, they may feel guilty or anxious about retiring early.

Moreover, social comparison can influence the amount of retirement savings an individual thinks they need, leading to anxiety about financial insecurity in retirement [52]. For example, individuals might compare their retirement savings with others who have a higher retirement fund. As a result, they may feel that their savings could be improved and worry about having insufficient money to support themselves in retirement.

Social comparison can contribute to retirement anxiety by creating unrealistic expectations, influencing retirement decisions, and affecting social support networks. This study aims to establish the essential impact of social comparison on the relationship between proactive behaviour and anxieties associated with retirement and proffer suggestions for better retirement preparation. 

**Figure 1:**
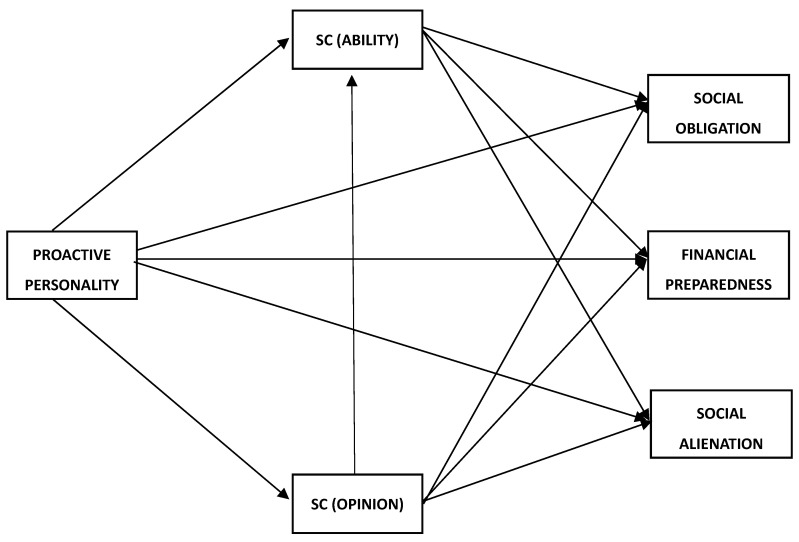
Conceptual model.

In conclusion, social comparison can facilitate the relationship between proactive behaviour and retirement anxiety by creating idealistic hopes and increasing unfavourable social comparisons. Therefore, it is essential to recognise the impact of social comparison and develop strategies to address any anxieties or concerns about retirement early enough. This may involve seeking support from family, friends, or a mental health professional, developing realistic retirement plans, and building a supportive social network of retired individuals (see Figure 1).

**H3.**  *Mediating role of social comparison (ability and opinion) in the relationship between proactive personality and retirement anxiety.*

## 4. Method

### 4.1. Participants

Participants in this study comprised 508 civil servants from tertiary institutions in the state capitals of six states. Using a table of random numbers from SPSS version 28, one state was selected from each of the six geopolitical zones of Nigeria. Participants were purposively selected from government-owned tertiary institutions in the states’ capitals. Participants who had five years or less of working life before retirement were the target population because thoughts about retirement thoughts could be of concern within this period. Of the total sample, 268 (52.8%) were males. Their mean age was 57.47 (SD = 3.02), ranging from 55 to 69 years. The majority of the participants were married, coming to a total of 414 (81.5%) married participants. 

### 4.2. Material 

#### 4.2.1. Proactive Personality

The proactive personality scale created by [53] was used to measure proactive personality. This 10-item scale evaluated an individual’s efforts to navigate a challenging work environment by seeking and creating opportunities for positive outcomes. For example, some of the questions asked respondents to rate their level of agreement with statements such as “I am constantly searching for new ways to improve my life” and “I believe that I can make things happen even in the face of adversity”. In addition, respondents were asked to indicate their level of agreement or disagreement on a five-point Likert scale ranging from strongly disagree (1) to strongly agree (5). Higher scores on the scale indicated a greater degree of proactive personality, while lower scores indicated a lower proactive personality. 

#### 4.2.2. Social Comparison Order

The social comparison orientation scale [54] was adapted to measure how future retirees compare themselves to their peers in the present study. This instrument measures the inclination to engage in social comparison and encompasses the self, the other, and the psychological relationship between them. The scale contains statements such as “I often compare what I have with others”, “I have done better than my relations”, and “I think about those better than me when making decisions”. Some statements were modified and approved by the original authors; for example, item four was changed from “I often compare how I am doing socially (e.g., social skills, popularity) with other people” to “I consider my situation in life relative to that of other people who started with me”. The scale comprises two dimensions, ability and opinion, with ability measuring the comparison of an individual’s abilities with others (items 1–6) and opinion measuring comparisons of individuals’ opinions with others (items 7–11). The 11-item instrument uses a five-point scale, ranging from 1 (strongly disagree) to 5 (strongly agree), with two items (5 and 11) being reverse scored. 

#### 4.2.3. Retirement Anxiety 

The Nigerian pre-retirement anxiety scale (NPAS) developed by [22] was utilised to evaluate the retirement anxiety of potential retirees. This scale consists of 15 items rated on a five-point scale ranging from 1 (strongly disagree) to 5 (strongly agree). The respondents indicated the degree to which they agreed with each item concerning their current level of retirement anxiety, with items 8, 9, 10, 11, and 14 being reverse scored. Higher scores on the NPAS indicate higher anxiety about retirement, while lower scores indicate lower anxiety about retirement. The NPAS has three dimensions, including a six-item social obligation section that deals with essential duties expected from an individual due to their status. An example of an item in this section is, “I feel I have not provided for my basic necessities before my retirement (housing, car, etc.)”. There is also a five-item financial preparedness section that deals with financial savings and investments. For instance, an example of an item in this section is “I have some reliable source of income after retirement to meet my lifestyle”. Finally, a four-item social alienation section deals with the fear of being neglected and becoming irrelevant in the family or society. An example of an item in this section is, “I am afraid I will be lonely when I retire”.

#### 4.2.4. Procedure 

The sampling strategy aimed to cover Nigeria as a nation, and one state from each of the six geopolitical zones of Nigeria was sampled. Six research assistants, including civil servants, lecturers, and students familiar with field data collection procedures, were recruited and trained to distribute questionnaires in the six surveyed states. They assisted with distributing and collecting 600 copies of the questionnaire over two months, with 100 copies distributed in each state. The primary inclusion criterion was that the participants were within five years of retirement. The rationale for excluding participants with more than five years to retirement was based on research indicating that individuals closer to retirement tend to be more concerned about it [22,55]. The participants were informed that their participation was voluntary and the data collected would remain confidential. The questionnaire could be completed in approximately 10 min. Out of the 600 copies distributed, only 524 copies were returned. After cleaning the data, 508 valid copies were used for analysis (see Table 1). The valid response rate was 56.44%. 

#### 4.2.5. Design and Statistics

The researchers adopted a cross-sectional research design. Pearson correlation statistics were conducted using SPSS version 28, while the measurement models and mediation tests were conducted using IBM AMOS version 28.

## 5. Measurement Model

The internal consistency reliability, convergent validity, and discriminant validity of the measurement model were evaluated in the study [56,57]. Table 2 displays the results for internal consistency reliability, and all Cronbach’s alphas and composite reliability values exceeded the minimum required value of 0.70, indicating acceptable reliabilities for all measures used.

Convergent validity was confirmed by examining the outer loadings of individual indicators and the average variance extracted (AVE) in retirement anxiety factors, social comparison, and proactive personality [56,57]. The AVE values for these constructs all exceeded the required minimum of 0.50, demonstrating convergent validity for the measurement model.

Discriminant validity was evaluated using the Fornell–Larcker criterion and the HTMT ratio of correlations [58]. The Fornell–Larcker criterion was met, as the square root of each corresponding AVE was greater than the correlations between it and other constructs. The HTMT ratio of correlations was less than 0.85 for all measures, indicating that discriminant validity was established [59]. The bootstrap confidence intervals further supported discriminant validity, as none included the value 1. The off-diagonal elements in the correlation coefficients between constructs were smaller than the diagonal elements, indicating that discriminant validity was met for all measures (see Table 3 and Table 4).

Therefore, the measurement model demonstrated acceptable internal consistency reliability, convergent validity, and discriminant validity, indicating that the measures used in the study were reliable and valid for measuring retirement anxiety factors, social comparison, and proactive personality.

The results of the correlations in Table 4 indicate that except for educational level, all other demographic variables entered as control significantly correlated with a dimension of retirement anxiety (social obligation, financial preparedness, and social alienation). Thus, following Becker’s [60] recommendation, educational level was excluded when testing the hypotheses because of its insignificant correlation with retirement anxiety. Specifically, age (r = 0.13, *p* < 0.001; r = 0.10, *p* < 0.001) was positively related to social obligation and social alienation, respectively. The number of children (r = −0.08, *p* = 0.04; r = −0.07, and *p* = 0.044) was negatively related to social obligation and social alienation, respectively. Financial dependants (r = 0.09, *p* = 0.03) were positively related to social obligation. Proactive personality (r = −0.16, *p* < 0.001; r = −0.20, *p* < 0.001; and r = −0.14, *p* < 0.001) was negatively related to the three dimensions of retirement anxiety: social obligation, financial preparedness, and social alienation. Social comparison (ability) (r = 0.12, *p* < 0.001; r = 0.18, *p* < 0.001) was positively related to retirement anxiety dimensions (social obligation and social alienation), respectively. Social comparison (opinion) (r = 0.12, *p* < 0.001; r = 0.15, *p* < 0.001; and r = 0.25, *p* < 0.001) was positively related to all the dimensions of retirement anxiety (social obligation, financial preparedness, and social alienation), respectively.

## 6. Structural Equation Modelling 

The researchers used structural equation modelling (SEM) with manifest variables to test the mediation hypotheses. The goodness-of-fit of the models was assessed using several indices, including the Chi-square test, comparative fit index (CFI), normed fit index (NFI), Tucker–Lewis index (TLI), goodness-of-fit statistic (GFI), and the root-mean-square error of approximation (RMSEA). The researchers considered a suitable model when the Chi-square value was non-significant, the TLI, CFI, NFI, and GFI were greater than 0.95, and the RMSEA was less than 0.05, following advice from [61,62,63]. The researchers used the square multiple correlations to determine how much of the variance in retirement anxiety was explained by the independent variables.

The researchers calculated total, direct, and indirect effects between the independent and dependent variables using standardised path coefficients and maximum likelihood estimation. SEM allowed the researchers to test direct and indirect effects simultaneously within the model. The researchers used the bias-corrected bootstrap method with 5000 iterations and a 95% confidence interval (CI) to test the indirect effects, as [64] recommended.

Before data collection, the researchers determined the sample size based on available data from previous studies, such as [65]. To detect a standardised difference with a power of 80% and a two-sided significance level of 0.05, the researchers needed a minimum of 56 subjects. The researchers adjusted the path model for age, gender, marital status, number of children, educational level, and financial dependents, following [66]. The researchers considered a *p*-value of 0.05 or less to be statistically significant. 

Three structural models were tested, first assessing the structural model while omitting both mediators (model 1). Secondly, a parallel mediation including the two mediators (model 2) was tested. Lastly, a sequential mediation to investigate the mediating effect of SCo and SCa between PP and retirement anxiety domains (model 3) was tested (see Table 5).

### Mediation Analysis

**Figure 2:**
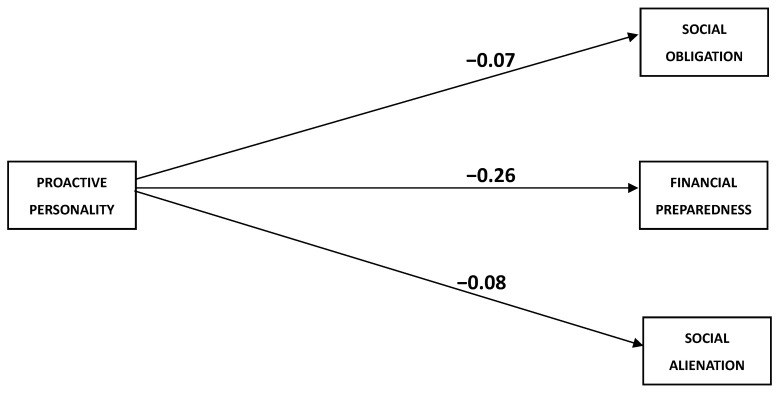
Direct effect of proactive personality and retirement anxiety domains.

**Figure 3:**
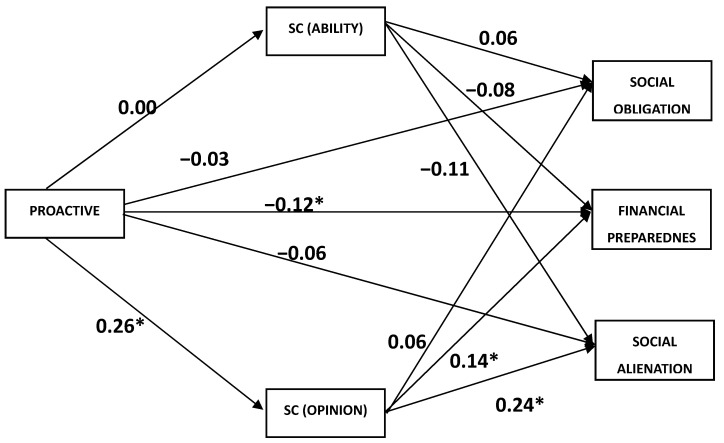
Mediating role of social comparison (ability and opinion) in the relationship between proactive personality and retirement anxiety domains. * *p* < 0.05.

Table 6 indicates the results from the 5000 bootstrapped samples measuring the mediating role of social comparison domains (ability and opinion) in the relationship between proactive personality and retirement anxiety (social obligation, financial preparedness, and social alienation) (see Table 6, Figure 2 and Figure 3).

Discussing the proactive personality (PP) of the first dimension of retirement anxiety (social obligation, SO) indicated that the total effect of PP on SO was significant (β_total_ = 0.076, SE = 0.037, *p* < 0.05), the direct effect (PP and SO) was also significant (β_direct_ = 0.047, SE = 0.043, *p* < 0.05), and the specific indirect effect (SIE) of social comparison (ability) (SCa, mediator) was not significant, indicating that SCa did not mediate the relationship. 

The other specific indirect effect through SCo and the sequential indirect effect through SCa and SCo were equally not significant mediators in the relationship between PP and SO.

The second dimension of retirement anxiety (financial preparedness, FP) showed that the total effect of PP on FP was significant (β_total_ = 0.259, SE = 0.048, and *p* = 0.001). The direct effect of PP and FP was also significant (β_direct_ = 0.219, SE = 0.046, and *p* = 0.001). In contrast, the specific indirect effect through SCa included zero (SIE = −0.001, 95% CI: LL = −0.004 to UL = 0.001), indicating the absence of a mediation effect. The second specific indirect effect through SCo was significant and did not include zero (SIE = 0.035, 95% CI: LL = 0.022 to UL = 0.052), indicating the presence of partial mediation. The third specific indirect effect through SCo and SCa was equally significant (SIE = −0.018, 95% CI: LL = −0.028 to UL = −0.009), indicating the presence of a sequential partial mediation.

In the third dimension of retirement anxiety (social alienation, SA), the total effect of PP on SA was significant (β_total_ = 0.098, SE = 0.049, *p* < 0.05), and the direct effect of PP and SA was significant (β_direct_ = 0.015, SE = 0.038, *p* < 0.05). Still, the specific indirect effect through SCa was not significant (SIE = 0.000, 95% CI: LL = −0.003 to UL = 0.001), indicating the absence of mediation. On the other hand, the second specific indirect effect through SCo was significant and did not include zero (SIE = 0.044, 95% CI: LL = 0.010 to UL = 0.026), indicating the presence of partial mediation. Finally, the last sequential mediation through SCo and SCa was not significant (SIE = −0.008, 95% CI: LL = −0.021 to UL = 0.005), indicating the absence of mediation.

## 7. Discussion

The present study sought to examine the mediating role of social comparison in the relationship between proactive personality and retirement anxiety in Nigerian civil servants. 

The reviewed literature reveals that retirement anxiety arises due to the apprehension of adjusting to a new lifestyle and the fear of running out of money, especially for those who relied on their career as a primary source of income, identity, and purpose. The review noted that Nigeria adopted the contributory pension scheme in 2004, which shifted the risk of retirement savings into the hands of the employee. However, low savings, volatile financial markets, rapid population growth, and lack of political will make it difficult for civil servants to feel confident about their financial futures. The results of this study resonate with those previously conducted on the subject [24,25,63]. The result supports H1, that a proactive personality negatively predicted retirement anxiety—the three domains of retirement anxiety are social obligation, financial preparedness, and social alienation. The findings support that the fear of not meeting one’s obligations or maintaining a lifestyle could be challenging once one retires [65]. Inadequate financial status to sustain them for years after retirement might not be a reality, nor may reliance on their children or family for financial support be a reality [2,9,23]. Additionally, the fear of being isolated from all their previously secured social networks would soon happen, as they might lose their supposed value or relevance after retirement [9].

This finding points to the fact that civil servants are aware that their pension would not sustain their retirement lives, and as a result, they seek ways to augment their savings by engaging in diverse forms of intrapreneurship/entrepreneurship within and outside their regular work schedules [19]. This could explain the prevalence of diverse forms of small businesses in Nigerian work places [17,18].

The second hypothesis that postulated that proactive personality would predict social comparison (ability and opinion) was partly supported. The relationship was positively related to social comparison (opinion), but not social comparison (ability). The result is in line with previous studies (e.g., [20,42,43,44]) that established that the choices to save or invest are made relative to people and status in society, where low-income people are more likely to save or invest based on the information around them. The less information people have, the less likely they are to invest or save. Moreover, people rely on other people’s opinions on financial decisions rather than their own investigation (e.g., [67]). This reflects the collectivistic mentality of African society, where ideas are collectively considered acceptable and followed as a way of life. This is reflected in Nigeria where people, especially parents, invest in their family and children, expecting that the family and children will take care of them after retirement rather than engaging in other concrete forms of investment or saving for retirement. These uncertainties of life induce anxiety as people approach retirement. 

The third hypothesis, which states that social comparison (ability and opinion) would mediate the relationship between proactive personality and retirement anxiety, was partly supported. The result reveals that social comparison (opinion) mediated the relationship between proactive personality and retirement anxiety (financial preparedness and social alienation). Furthermore, social comparison (opinion and ability) mediated the relationship between proactive personality and retirement anxiety (financial preparedness) in sequential order. This result concurs with the social comparison theory (Festinger, 1957 [46]), which opines that decision-making does not happen in isolation. The constant comparison with others keeps most societies predictable. Social comparison studies over the years showed both positive and negative effects on people. Its influence on retirement anxiety points to the expectations, which in most cases are unrealistic outcomes of people’s investment strategies for retirement. The first part of the hypothesis showed that social comparison (opinion) mediated the relationship between proactive behaviour and retirement anxiety (financial preparedness), and revealed that as much as it was established that proactive behaviour reduces retirement anxiety, social comparison (opinion) plays a significant facilitating role in the financial preparedness of an individual. The financial decisions people make rely more heavily on the opinions of others than on facts. Therefore, the financial opinions of those around an individual significantly influence his choices. 

The second part of the hypothesis indicates that social comparison (opinion) mediated the relationship between proactive personality and retirement anxiety (social alienation). This finding is supported by some earlier studies (e.g., [68]). The fear of being isolated and irrelevant could trigger anxiety. The desire to be valued and have meaning in one’s life seems transient with a predetermined date. 

Finally, the result shows that social comparison and sequential mediation of opinion as well as ability influenced the relationship between proactive personality and retirement anxiety (financial preparedness). The result shows that linking opinion and ability creates a chain of effects. Each mediator (opinion and ability) affects the next in the chain, ultimately leading to retirement anxiety (financial preparedness). 

The novelty of this result further shows the complexity of the challenges retirees face. Highly proactive behaviour could lead to an upward social comparison of opinions, considering the opinion of those they feel are better than them. This leads to an upward social comparison of ability. This means seeking to outclass those better than them, consequently engaging in behaviour that will reduce their fear of financial inadequacies because they carried out things that prepare them better financially for retirement. 

## 8. Implications of the Study

The study has several practical implications for policymakers, employers, and employees in Nigeria.

Firstly, the finding that retirement anxiety is prevalent among civil servants and that their pensions may not be sufficient to sustain their retirement years highlights the need for policymakers to review the current pension scheme in Nigeria. They could consider developing policies and programmes that would increase the retirement benefits of civil servants in the education sector to help them feel more financially secure and less anxious about their retirement years.

Secondly, the outcome that civil servants engage in diverse forms of intrapreneurship/entrepreneurship within and outside their regular work schedules to augment their savings underscores the need for employers to support their employees’ entrepreneurial activities. Employers could provide resources and training to help employees convert their research to businesses while still in service, thus improving employees’ job commitment and also helping them save more for retirement.

Thirdly, the study’s findings suggest that social comparison plays a significant role in retirement anxiety. Thus, there is a need for financial institutions to redesign and develop new products that would improve financial literacy and provide reliable services that build trust in their customers. This will enable people to make informed financial decisions, save and invest wisely, and avoid unfavourable social comparisons (consequent of others’ bad experiences) that can lead to anxiety.

Fourthly, the result that a proactive personality is negatively related to retirement anxiety highlights the need for individuals to cultivate proactive behaviours and attitudes towards retirement planning. For example, individuals could take proactive steps, such as seeking financial advice, developing multiple income streams, and learning new skills that could help them feel more confident and less anxious about retirement.

Finally, retirement is inevitable, and so is ageing. Finding meaning in life and lasting relationships with friends and family will be a strong buffer during ageing, leading to understanding that work life will pass, but life itself continues afterwards. 

## 9. Limitations

This study has limitations. One limitation of this study is the sampling strategy. The study used a simple random sampling technique to select one state from each of the six geopolitical zones of Nigeria and then purposively selected participants who had five years or less to retire from government-owned tertiary institutions in the selected state capitals. However, this sampling method may only represent part of Nigeria’s potential retirees. For instance, the sample only included civil servants from government-owned tertiary institutions. As such, it may not represent potential retirees in the private sector or other industries. 

Additionally, using self-reported measures, such as the proactive personality scale and the general pre-retirement anxiety scale may be subject to response bias and social desirability bias. Participants may be reluctant to report negative attitudes or feelings towards retirement, which could affect the results’ validity. Additionally, the study’s cross-sectional design limits the causal conclusions that can be drawn from the observed associations between variables. Finally, future research can be pursued on this topic still within the educational sector in other regions or other geographical settings to compare the findings. 

## 10. Conclusions 

This study explores the connection between proactive personality, social comparison, and retirement anxiety among Nigerian civil servants. It emphasises the significant impact of retirement anxiety due to concerns such as insufficient savings, financial instability, and potential loss of social networks post-retirement. The research highlights the role of social comparison, both in terms of opinions and abilities, in influencing financial decisions and alleviating retirement anxiety. The findings offer valuable insights for policymakers, employers, and individuals to encourage financial preparedness and minimise retirement anxiety in Nigeria. Lastly, the study calls for more research to understand better the complexities of retirement anxiety and the factors affecting financial decision-making among retirees.

## Figures and Tables

**Table 1:** Sample Demographics.

Variable	Categories	Frequency	Percent
Gender	Female	240	47.2
	Male	268	52.8
Marital Status	Single	32	6.3
	Married	414	81.5
	Divorced	18	3.5
	Widowed	44	8.7
Educational level	FSLC	58	11.4
	WAEC	58	11.4
	OND	24	4.7
	HND/BSC	174	34.3
	Postgraduate	194	38.2

**Table 2:** Reliability and convergent validity.

Variable	Indicator	Loading	CA	CR	AVE
SO	PR1	0.693	0.758	0.819	0.538
	PR2	0.749			
	PR3	0.786			
	PR4	0.620			
	PR5	0.859			
	PR6	0.702			
FP	PR7	0.940	0.804	0.775	0.532
	PR8	0.761			
	PR9	0.620			
	PR10	0.533			
	PR11	0.579			
SA	PR12	0.679	0.718	0.768	0.568
	PR13	0.590			
	PR14	0.840			
	PR15	0.842			
SC ability	SC1	0.716	0.890	0.910	0.630
	SC2	0.769			
	SC3	0.903			
	SC4	0.840			
	SC5	0.773			
	SC6	0.744			
SC opinion	SC7	0.758	0.846	0.880	0.597
	SC8	0.854			
	SC9	0.781			
	SC10	0.625			
	SC11	0.825			
Proactive personality	PP1	0.813	0.920	0.933	0.584
	PP2	0.687			
	PP3	0.801			
	PP4	0.850			
	PP5	0.725			
	PP6	0.818			
	PP7	0.560			
	PP8	0.779			
	PP9	0.876			
	PP10	0.681			

Note: AVE = average variance extracted, CA = Cronbach’s alpha, CR = composite reliability; SO = social obligation; FP = financial preparedness; SA = social alienation; and SC = social comparison.

**Table 3:** Discriminant validity HTMT (HTMT ratio of correlations).

	FP	Proactive Personality	SA	SC Ability	Sc Opinion	SO
FP						
Proactive personality	0.355					
SA	0.253	0.398				
SC (ability)	0.199	0.450	0.407			
SC (opinion)	0.278	0.533	0.507	0.800		
SO	0.287	0.283	0.799	0.228	0.247	

Note: SO = social obligation; FP = financial preparedness; SA = social alienation; and SC = social comparison.

**Table 4:** Descriptive, correlation matrix, and discriminant validity (Fornell–Larcker criterion).

	Variables	M	SD	1	2	3	4	5	6	7	8	9	10	11	12
1	Gender	−	−	1											
2	Age	57.47	3.02	−0.01	1										
3	Marital status	−	−	−0.06	0.34 **	1									
4	EL	−	−	0.12 **	0.51 **	0.27 **	1								
5	NC	2.79	3.10	0.09 **	0.45 **	0.22 **	0.36 **	1							
6	FD	2.45	2.95	0.06	0.35 **	0.14 **	0.35 **	0.69 **	1						
7	Proactive Personality	37.11	8.77	−0.10 **	0.37 **	0.05	0.15 **	−0.02	0.05	0.764					
8	SC (ability)	18.48	6.31	−0.12 **	0.19 **	−0.04	0.09 **	−0.03	−0.05	0.30 **	0.794				
9	SC (opinion)	15.71	5.22	−0.09 **	0.27 **	0.02	0.17 **	0.01	−0.08 *	0.38 **	0.77 **	0.773			
10	Social obligation	15.68	5.58	−0.14 **	0.13 **	0.01	0.02	−0.08 *	0.09 *	−0.16 **	0.12 **	0.12 **	0.734		
11	Financial preparedness	12.48	3.48	−0.04	0.03	0.18 **	−0.06	0.04	−0.02	−0.20 **	0.04	0.15 **	−0.06	0.729	
12	Social alienation	12.83	3.75	−0.02	0.10 **	−0.09 *	0.03	−0.07 *	-0.06	−0.14 **	0.18 **	0.25 **	0.43 **	0.27 **	0.754

Note: * *p* < 0.05, ** *p* < 0.01; italicized text represents Fornell–Larcker criterion; gender (dummy coded ‘1’ = male and ‘0’ = females); marital status (coded 1—single, 2—married, 3—divorced, and 4—widowed); EL = educational level (1—FSLC, 2—WAEC, 3—OND, 4—HND/BSC, 5—postgraduates); NC = number of children; FD = financial dependants; and SC = social comparison.

**Table 5:** Structural equation modelling.

Models	CMIN/df	RMR	CFI	NFI	TLI	GFI	RMSEA
Model 1	9.983	0.560	0.997	0.995	0.966	0.998	0.041
Model 2	28.966	0.609	0.994	0.991	0.964	0.995	0.042
Model 3	29.207	0.733	0.994	0.991	0.969	0.995	0.040

**Table 6:** Mediation of social comparison in the relationship between proactive personality and retirement anxiety.

Type of Effect		Estimate	SE	BCa 95%CI [LLCI, ULCI]		R^2^	Remark
SO as the DV							
Total Effect	PP→SO	0.076 *	0.037	0.001	0.148	0.093	
Direct effect	PP→SO	0.047 *	0.043	−0.029	0.121		
SIE	PP→SCa→SO	0.000	0.000	−0.001	0.003		No mediated
SIE	PP→SCo→SO	0.013	0.014	−0.015	0.041		No mediated
SIE	PP→SCo→SCa→SO	0.005	0.007	−0.011	0.023		No mediated
FP as the DV							
Total Effect	PP→FP	0.259 **	0.048	0.160	0.348	0.155	
Direct effect	PP→FP	0.219 **	0.046	0.124	0.304		
SIE	PP→SCa→FP	−0.001	0.001	−0.004	0.001		No mediated
SIE	PP→SCo→FP	0.035 **	0.008	0.022	0.052		mediated
SIE	PP→SCo→SCa→FP	−0.018 **	0.005	−0.028	−0.009		mediated
SA as the DV							
Total Effect	PP→SA	0.098 *	0.049	0.001	0.192	0.076	
Direct effect	PP→SA	0.015 *	0.038	−0.073	0.094		
SIE	PP→SCa→SA	0.000	0.001	−0.003	0.001		No mediated
SIE	PP→Sco→SA	0.044 **	0.010	0.026	0.064		mediated
SIE	PP→SCo→SCa→SA	−0.008	0.006	−0.021	0.005		No mediated

Note: * *p* < 0.05, ** *p* < 0.001; DV = dependent variable; SIE = specific indirect effect; PP = proactive personality; SCa = social comparison (ability); SCo = social comparison (opinion); SO = social obligation; FP = financial preparedness; and SA = social alienation.

## Data Availability

The datasets generated during and/or analysed during the current study are available from the corresponding author on reasonable request.
